# Reporting epidemics: newspapers, information dissemination and the story of Ebola in the Ugandan district of Luweero

**DOI:** 10.4314/pamj.v9i1.71222

**Published:** 2011-08-23

**Authors:** Allan Mwesiga

**Affiliations:** 1 Pan African Medical Journal, African Field Epidemiology Network (AFENET), Kampala, Uganda

**Keywords:** Ebola, Uganda, epidemic, case, reporting, media, Uganda

## Abstract

When an outbreak occurs, the affected population needs timely information in order to make informed decisions on how best to deal with the situation. Most target populations rely on the media for their information and the authorities use the media to disseminate outbreak information. The media, particularly locally based media, is as a result, crucial to public health outcomes. Reports on outbreaks should be as easy to understand as possible. However, there is, at times, a mismatch between the ideal and the practice. In looking at an example of the practice, this opinion hopes to influence the negotiation for the ideal in outbreak reporting.

## Opinion

Headlines can be dramatic. Take two from Uganda's leading dailies on the 15^th^ of May 2011, the day after Uganda's Ministry of Health made notice of an Ebola outbreak. The New Vision runs a story with the headline: “Health team visits Ebola-hit Luweero” [1]. The Daily Monitor took a more shrill tone: “Ebola Virus breaks out in Uganda” [2].

The basic facts were not as dramatic. A 12-year old girl had died eight days earlier at Bombo Military Hospital of what was confirmed, by laboratory tests at the Uganda Virus Research Institute, to have been Ebola.

That was the basic story run by AFP [3], Reuters [4], and Xinhua [5] on the 14^th^ and 15^th^ (Xinhua) of May 2011. The Wall Street Journal, four days later took the rather unusual angle of suggesting it could have been an isolated case [6]. The World Health Organisation had an item in its Disease Outbreak News with a more clinical orientation [7]. The US based Centers for Disease Control and Prevention (CDC) had an outbreak posting that mentioned how the diagnosis was confirmed and the technical assistance being provided to assist in outbreak response [8].

Uganda has had three outbreaks of Ebola Hemorrhagic Fever (EHF). The first was in Gulu in the north (2000) [9], the second in Bundibudgyo in the west (2007) [10] and the latest in Luweero was in the Central region [11]. With early symptoms akin to a febrile illness, a causative agent named after a river in the Democratic Republic of Congo and no cure, Ebola is a touchy subject to communicate. A suspected case in Mityana in 2009 was reported to have triggered a panic [12]. The article used the term “suspected outbreak” to describe a situation where a woman presenting with abdominal pain, vomiting and bleeding was quarantined [13]. Only months before the Ministry of Health had held a media sensitization workshop on epidemics [14]. Later in the year more even handed language was used to describe a man with symptoms of a febrile illness admitted to Mbale Hospital in the east of the country [15].

Six days after the initial announcement of the latest outbreak in Luweero, the coverage turned dramatic. The New Vision reported that two cases had been “detected” in the districts of Nakaseke and Luweero [16]. The article strongly suggests a difference between a “detected” case and a “confirmed” case of Ebola going as far as calling the two patients “victims” [17]. The Daily Monitor used the term “new suspected case” and cited the Ministry of Health in its report about a girl admitted to Bugiri Hospital with symptoms of hemorrhagic fever [18]. A man admitted to Kagando Hospital in Kasese was described as having the “signs of the disease” an applaudable highlight that was spoiled by the mention of the latest Ebola outbreak as being the “third outbreak of the deadly disease in as many years” [19].

Going by media accounts alone, there existed, six days after outbreak confirmation, three “suspected cases” of Ebola with none confirmed. A particularly strident editorial on the 22 May 2011 urged for additional government facilitation to enable the investigation of “suspicious Ebola cases” [20].

May 24^th^ [21] and May 26^th^ [22] saw better coverage though the language left a lot to be desired. Both articles were still trying to justify using the word “case” without a confirmatory bias. This in a situation where easily accessible information was required by a public clearly concerned by the disease.

A lot of useful information about symptoms and control measures made it to print. What was conspicuously absent was the clarity; in using “confirmed” and “suspected”, it was still not clear how many-actual cases-of Ebola there were. Instead the uncertainty continued with a number of suspected and unconfirmed cases. In early June the coverage focused on analysis and the announcement of the end of the epidemic with no additional cases confirmed [23–25].

There is need for more clarity in the way outbreaks are reported. Training opportunities abound and closer collaboration with the media through quarterly media sensitization workshops on epidemics would provide a much needed critical mass of public health aware journalists as well as contacts between media and other health actors.

Guidelines for reporting on epidemics can be drawn and easy to define terms agreed upon so that cases do not always have to be qualified in cluttered phrases likely to be misunderstood by a public rightly alarmed but grossly short of relevant information.

## References

[1] Baguma Raymond Health team visits Ebola-hit Luweero. The New Vision.

[2] Lirri Evelyn Ebola virus breaks out in Uganda. The Daily Monitor.

[3] AFP Ebola virus case reported near Uganda's capital. http://www.google.com/hostednews/afp/article/ALeqM5jW5iN9-ovtxusI0A9jKNTiQQd_ZA?docId=CNG.99d7eac3ff8a824197cae4070ca6f8e1.671.

[4] Malone Barry Ebola Kills Girl in Uganda with More Cases Expected-Reuters. http://www.reuters.com/article/2011/05/14/us-uganda-ebola-idUSTRE74D12220110514.

[5] Deadly Ebola breaks out in Uganda, kills one, 30 monitored English.news.cn. http://news.xinhuanet.com/english2010/health/2011-05/15/c_13875099.htm.

[6] Connors Will Ebola case spurs concern in Uganda Wall Street Journal online. http://online.wsj.com/article/SB10001424052748703509104576329651658268930.html.

[7] Ebola in Uganda. WHO.

[8] Outbreak Postings-CDC Special Pathogens Branch. Centers for Disease Control and Prevention.

[9] MMWR Morb Mortal Wkly Rep

[10] WHO (2007). Ebola haemorrhagic fever in Uganda-update. WHO.

[11] Outbreak news (2011). Ebola, Uganda. Wkly Epidemiol Rec.

[12] Luke Kagiri Suspected Ebola Patient in Mityana. The New Vision.

[13] Luke Kagiri Suspected Ebola Patient in Mityana. The New Vision.

[14] Anthony Bugembe (2011). The New Vision. Available at http://www.newvision.co.ug/D/8/13/615329/ebola%20outbreak.

[15] Anne Mugisa, Baguma Raymond Tests Rule out Ebola in Mbale. The New Vision.

[16] Henry Sekanjako, Nabatanzi Violet Two More Ebola Cases Detected. The New Vision.

[17] Henry Sekanjako, Nabatanzi Violet Two More Ebola Cases Detected. The New Vision.

[18] Dan Wandera Suspected Ebola Case in Bugiri. The Daily Monitor.

[19] Flavia Lanyero Two Deaths in Luweero Linked to Ebola. The Daily Monitor.

[20] Give Ebola Outbreak Countrywide Alert. The Daily Monitor.

[21] Flavia Lanyero Health Experts Call for Calm as New Ebola Cases Are Suspected. The Daily Monitor.

[22] Violet Nabatanzi, Baguma Raymond Ebola Outbreak under Control. The New Vision.

[23] Philippa Croome Is Ebola an Emerging Pandemic?. The Daily Monitor.

[24] Mary Karugaba, Anguyo Innocent Uganda Declared Free of Ebola. The Daily Monitor.

[25] Evelyn Lirri Uganda Declared Ebola-free Again. The Daily Monitor.

